# The Effects of Suicide and Homicide on Clinicians

**DOI:** 10.1192/bjo.2025.10363

**Published:** 2025-06-20

**Authors:** Christiana Elisha-Aboh, Hany El-Sayeh, Rachel Gibbons

**Affiliations:** 1 Tees, Esk and Wear Valley Foundation Trust, York, United Kingdom; 2 Tees, Esk and Wear Valley Foundation Trust, North Allerton, United Kingdom.; 3 Independent Researcher, London, United Kingdom

## Abstract

**Aims:** The effects on professionals following the death of a patient by suicide can be phenomenal and life changing. The Royal College of Psychiatrists has developed guidelines to promote operational strategies and adequate pastoral care for professionals affected by patient suicides. Recognizing the profound impact on mental health, burnout, retention and career progression, these guidelines aim to foster a supportive culture. Enhanced support could facilitate genuine reflection and learning from such incidents, ultimately leading to improved patient care.

The aim was to discuss the impact of suicides and homicides on clinicians while exploring available support structures and understanding relevant psychological processes.

**Methods:** On October 25, 2024, a one-hour medical webinar hosted 87 participants, including doctors, medical students, and nursing staff. Led by Dr Rachel Gibbons, an experienced consultant psychiatrist, the session focused on clinician vulnerabilities and defensive mechanisms. Pre- and post-workshop surveys evaluated areas of interest and effectiveness for future planning.

**Results:** The pre-survey results revealed that 34% of respondents were primarily interested in the potential blame associated with incidents, while 16% sought guidance on supporting colleagues. Notably, 65% had experienced a Serious Untoward Incident (SUI), predominantly suicides and homicides (92%), with many professionals expressing self-blame and feelings of failure. They struggled to support affected families and felt the review process often emphasized blame rather than learning.

In the post-survey, 77% of responders reported involvement in an SUI, with 88% linked to suicides or homicides. Support perceptions varied: 36% felt supported by fellow doctors, and 20% by their trust, while colleagues (52%) and family and friends (56%) were highlighted as key sources of support. Most learned about incidents through emails, phone calls, or word of mouth (64%), and only 40% were satisfied with how they were informed. Respondents emphasized the importance of sensitive communication and individualized support plans in enhancing their experiences.

**Conclusion:** Overall feedback was overwhelmingly positive, with 93% of attendees expressing interest in future events. An impressive 97% found the seminar very or extremely helpful, while 93% wanted webinars on supporting clinicians, bereaved families, and attending coroner’s court. Many reported significant emotional impacts from suicides, affecting performance in 41% and prompting 27% to consider leaving psychiatry. Attendees emphasized the need for better support systems, compassionate communication, and debriefs to alleviate blame culture and improve coping with immediate effects.

Upcoming webinars will utilise feedback, ensure wider participation, engage senior management, and raise awareness of pastoral support strategies.

